# A Subdural Bioelectronic Implant to Record Electrical Activity from the Spinal Cord in Freely Moving Rats

**DOI:** 10.1002/advs.202105913

**Published:** 2022-05-02

**Authors:** Bruce Harland, Zaid Aqrawe, Maria Vomero, Christian Boehler, Ernest Cheah, Brad Raos, Maria Asplund, Simon J. O'Carroll, Darren Svirskis

**Affiliations:** ^1^ School of Pharmacy The University of Auckland Auckland 1023 New Zealand; ^2^ Department of Microsystems Engineering (IMTEK) BrainLinks‐BrainTools Center University of Freiburg Freiburg 79110 Germany; ^3^ Department of Microsystems Engineering (IMTEK) BrainLinks‐BrainTools Center and Freiburg Institute for Advanced Studies (FRIAS) University of Freiburg Freiburg 79110 Germany; ^4^ Division of Nursing and Medical Technology Luleå University of Technology Luleå 971 87 Sweden; ^5^ Department of Anatomy & Medical Imaging School of Medical Sciences The University of Auckland Auckland 1023 New Zealand

**Keywords:** bioelectronic implant, electroceutical, polyimide, spinal cord implant, spinal cord injury, spinal recording

## Abstract

Bioelectronic devices have found use at the interface with neural tissue to investigate and treat nervous system disorders. Here, the development and characterization of a very thin flexible bioelectronic implant inserted along the thoracic spinal cord in rats directly in contact with and conformable to the dorsal surface of the spinal cord are presented. There is no negative impact on hind‐limb functionality nor any change in the volume or shape of the spinal cord. The bioelectronic implant is maintained in rats for a period of 12 weeks. The first subdural recordings of spinal cord activity in freely moving animals are presented; rats are plugged in via a recording cable and allowed to freely behave and move around on a raised platform. Recordings contained multiple distinct voltage waveforms spatially localize to individual electrodes. This device has great potential to monitor electrical signaling in the spinal cord after an injury and in the future, this implant will facilitate the identification of biomarkers in spinal cord injury and recovery, while enabling the delivery of localized electroceutical and chemical treatments.

## Graphical abstract

A very thin flexible bioelectronic implant that is inserted along the thoracic spinal cord in rats is demonstrated. Despite the highly sensitive environment, there is no negative impact on hind‐limb functionality over 12 weeks. The implant provides direct and continuous access to the spinal cord, allowing the first electrical recordings of spinal activity to be reported from freely behaving animals.

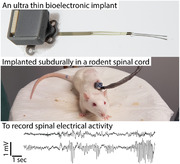

## 1 Introduction

Bioelectronic devices have made significant contributions to the treatment of deafness, paralysis, epilepsy, and blindness through electrical recording and targeted stimulation of electrically active nerves.^[^ ^1^, ^2^, ^3^, ^4^ ^]^ These devices are usually comprised of metallic electrodes which are patterned onto, and insulated by, flexible materials such as polyimide, parylene‐C or poly‐(dimethyl siloxane) (PDMS).^[^ ^5^, ^6^, ^7^ ^]^ The area of application and intended use defines the final device geometry, electrode dimensions, and materials used. Here we show for the first time a polyimide based bioelectronic implant compatible with the highly sensitive spinal cord environment, capable of monitoring spinal cord electrical activity. We maintained this subdural implant and its external connector for a 12 week period during which the implant provided direct and continuous access to the spinal cord, and report for the first time electrical recordings of spinal activity in freely behaving animals.

Polyimide and PDMS have both previously been described for use in contact with the spinal cord.^[^ ^8^, ^9^ ^]^ A PDMS spinal implant (termed the e‐dura) has been used to treat spinal cord injury (SCI) based on a different approach of neurostimulation to externally drive locomotor function in rats by bypassing the injury site.^[^ ^9^ ^]^ Targeted stimulation of the lumbosacral spinal cord during weight assisted treadmill rehabilitation led to improvements in locomotion during a 6‐week period in rats with SCI. This treatment approach has been successfully translated to a clinical setting, resulting in recovery of voluntary control of leg muscles, which in some cases persists even after electrical stimulation was turned off.^[^ ^10^ ^]^ The e‐dura, composed of the soft elastomer PDMS, was inserted below the dura mater enabling electrical stimulation at lower voltage than what is typically associated with epidural placement, and allowing direct access to the spinal cord for drug administration.^[^ ^10^ ^]^ Nevertheless, challenges related to elastomeric and soft implants have subsequently been described.^[^ ^11^ ^]^ One of the main challenges of elastomeric bioelectronics is that conductive connection lines and electrodes also have to be made stretchable to draw full advantage of the system. Stretchability comes at the cost of high conductivity, as this is linked to crystallinity of the conductor. Thus, to maintain reasonably low impedances in a stretchable connection line, the line has to be made substantially larger than a non‐stretchable one.

Compared to PDMS, major advantages of polyimide are that the photolithographic methods for microfabrication are well‐established and polyimide films can be made ultra‐thin—on the order of single micrometers. On polyimide, metallizations can be patterned at micrometer precision using lift‐off techniques, forming much smaller electrodes than is possible on PDMS. With probe contours defined by dry‐etching, polyimide devices can be made extremely compact, and still be durable and able to sustain long implantation periods.^[^ ^12^ ^]^ Recently, polyimide has been shown to be an effective material for use in sciatic nerve implants,^[^ ^13^ ^]^ and high resolution penetrating brain electrode devices suitable for brain machine interface.^[^ ^14^, ^15^ ^]^ Polyimide has also been used previously for epi‐ and intra‐cortical neural probes,^[^ ^12^, ^16^, ^17^, ^18^ ^]^ and epidural spinal implants.^[^ ^8^, ^19^ ^]^ However, it has previously been shown to be incompatible with subdural insertion along the spinal cord, with it resulting in a profound foreign body response, deformation of the spinal cord, and impaired motor function compared to the PDMS implant.^[^ ^9^ ^]^ While the Young's modulus of polyimide is far from the soft qualities of PDMS the biocompatibility of an implant should not be considered separately from its geometry. The use of ultra‐thin polyimide films has recently been demonstrated to achieve devices sufficiently compliant to conform to the contours of soft nervous tissue.^[^ ^16^ ^]^ Here, the biocompatibility disadvantages of polyimide are compensated for by true miniaturization of the implant meaning even the non‐stretchable polyimide should conform to the surface of the spine instead of compressing it. With these recent developments in mind, we here re‐explore the compatibility of an ultra‐thin polyimide implant with subdural insertion along the thoracic spinal cord.

We show that our polyimide based bioelectronic implant is capable of recording spinal cord electrical activity in awake freely moving animals. In the future, this will allow electrical activity to be characterized in non‐injured and SCI rats. This will increase the understanding of normal spinal cord electrical activity and changes that occur during injury and recovery. Meanwhile, the implant described can be augmented with dedicated stimulation electrodes to allow the administration of electroceutical treatments, either functional electrical stimulation for restoring mobility such as described by Squair et al.,^[^ ^20^ ^]^ or regenerative stimulation to support functional recovery. In vitro data, from cell based studies, indicate electrical stimulation can regenerate injured axons,^[^ ^21^, ^22^, ^23^ ^]^ an approach that holds great promise for treatment of SCI. A future bioelectronic implant that makes use of electrical recordings to “map” the boundaries of an injury site would have tremendous potential for use in a clinical setting, to detect the extent of injury and inform the location for delivery of personalized treatments.

To be an effective platform to probe spinal cord injury and deliver local electroceutical treatments, it is essential to first show that i) the implant causes no impairment of motor function, ii) has minimal effect on spinal cord shape and foreign body response, iii) that it can be implanted for sufficiently long to influence regeneration and then observe any resulting functional improvements after SCI,^[^ ^24^ ^]^ and iv) is capable of recording spinal cord electrical activity in an awake freely moving subject. Here, we describe the fabrication and characterization of two designs of the bioelectronic implant, a planar version, and a bifurcated version, where the latter reduces pressure on the central spinal vein to alleviate potential behavioral or structural deficits postimplantation. We show that i) animals implanted for up to 5 weeks were healthy with no hind‐limb impairment, ii) implantation over 1 week had minimal effect on spinal cord structural integrity and foreign body response, iii) an external housing for the implants plug connector attached to back muscle via sutures and surgical mesh, allowed us to maintain the implant over a clinically relevant period for SCI treatment of 12 weeks, and iv) our implant can record electrical activity, which are, to our knowledge, the first spinal cord subdural electrical recordings from awake freely moving animals.

## 2 Results

### 2.1 Electrochemical Characterization of the Bioelectronic Implant

**Figure 1:**
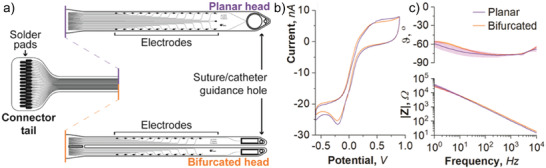
Two different configurations of the bioelectronic implant were designed and electrochemically characterized. a) The bioelectronic implants consisted of a connector tail which comprised solder pads, each connected to tracts running down the body of the implant. Two “head” designs were trialed and compared, being the planar head and bifurcated head. The platinum microelectrodes were characterized electrochemically in phosphate buffered saline through b) cyclic voltammetry (representative result plotted) and c) electrochemical impedance spectroscopy (*n* = 3 microelectrodes, data plotted as mean ± SD).

Polyimide based bioelectronic implants were fabricated at a thickness of 8 µm based on two designs, planar and bifurcated (**Figure** **1**a). Both designs had a total head width of 1.85 mm with 11 electrodes on each side, for a total of 22 electrodes, each with a diameter of 60 µm (electrodes numbered 1–11 on one side, and 17–27 on the other side (Figure 6)). The key difference between designs being the split down the middle of bifurcated devices was intended to reduce pressure on the central vein that runs along the dorsal midline of the spinal cord. Baseline electrochemical testing of the electrodes was undertaken in the form of cyclic voltammetry (CV) and electrochemical impedance spectroscopy (EIS) according to standard protocols.^[^ ^25^ ^]^ Representative CV scans of platinum microelectrodes embedded on either bifurcated or planar devices are displayed in Figure 1b. The scans show shapes that are typical of platinum microelectrodes (the same on planar and bifurcated devices) with reversible reduction and oxidation peaks at −0.3 and 0.4 V, respectively,^[^ ^26^ ^]^ likewise, the EIS analysis in Figure 1c displays spectra typical of planar metallic microelectrodes, where the impedance throughout the frequency range of interest (1 to 10000 Hz) is dominated by the double‐layer capacitance as visualized by a phase close to −90° and a slope on the |Z| versus frequency plot of −1.^[^ ^27^, ^28^ ^]^ The average impedance values at 1 kHz (|Z|_1kHz_) for the platinum microelectrodes on planar and bifurcated devices are 132 ± 9 kOhm and 107 ± 8 kOhm, respectively. These baseline characterization measures are important to assess the functionality of the entire system prior to implantation, from the implantable electrodes to the recording hardware.

### 2.2 In Vivo Implantation and Biocompatibility

**Figure 2:**
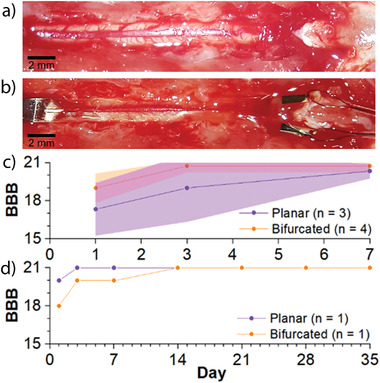
The bioelectronic implant had minimal impact on hind‐limb function for up to 5 weeks after subdural implantation in rats. a) Three spinal processes are removed (T12, T11, T10) and a section of spinal cord revealed. b) A bifurcated implant is shown; each arm has been inserted into the subdural space from the left side of the image, and the tips of each arm exit the subdural space on the right side of the image. c) Rats implanted with either version of the implant had only a transient loss of hind‐limb function and were generally unimpaired after a week (data reported as mean ± standard deviation). d) Two of the animals (1 with bifurcated, and 1 with planar implant) were monitored for 5 weeks after implantation and showed no longer‐term hind‐limb impairment or other physiological issues.

The ultimate goal of our study is to develop a bioelectronic device capable of recording and stimulating the injured spinal cord over time periods relevant for spinal cord regeneration. For this to be possible, we first need to show that implantation of the polyimide based bioelectronic device does not negatively impact the structural integrity of the spinal cord, or the locomotor function. Furthermore, the implant must be well tolerated by the tissue over time, inducing minimal foreign body response. A direct comparison was made across two designs, planar and bifurcated (Figure 1a), to investigate whether the presence of a bifurcated head designed to reduce pressure on the central vein influences behavioral or structural differences postimplantation. To study this, spinal processes T12, T11, and T10 were removed to expose the dorsal surface of the thoracic spinal cord (**Figure** **2**a) and planar or bifurcated implants were inserted subdurally (Figure 2b) in a cohort of rats for 1 week. This allowed us to score any initial injury caused by implantation and then see if any recovery occurred. Rats with even a moderate thoracic injury would be expected to show substantial recovery over the first week.^[^ ^24^ ^]^ Three different analyses were carried out (i) behavioral assessment of rats’ postimplantation using the Basso Beattie Bresnahan (BBB) scale, (ii) structural assessment of the spinal cord directly below the position of the bioelectronic implant and (iii) assessment of neuroinflammatory responses through the quantification of astrocytes and microglia in spinal tissue directly below the position of the bioelectronic implant. In these experiments, the bioelectronic head was inserted along the spinal cord and the body of the implant positioned subcutaneously but severed from the printed circuit board and plug connector, so that no parts of the implant remained external to the animal. This allowed the impact of the spinal implant to be assessed without the influence of additional forces associated with an external connector.

#### 2.2.1 Insertion of the Bioelectronic Implant Did Not Affect Hind‐Limb Function

Rats implanted with either planar or bifurcated devices displayed a mild transitory hind‐limb deficit over the first week, with no statistical difference noticed between the two different implants, as measured through the BBB scale (Figure 2c). In all rats, there was a noticeable improvement in BBB‐scores over time (Main Effect of Day; *F*(2,10) = 10.01, *p* < 0.01). Most recovery occurred by day 3 (Tukey's post hoc: day 1 vs day 3, *p* < 0.05), and this remained at day 7 (day 3 vs day 7, *p* = 0.56). There was no main effect of Group (*p* = 0.19) or interaction (Group*Day; *p *= 0.43) which suggested that there was no difference between the bifurcated and planar implants in terms of hind‐limb recovery. In both groups, animals were generally unimpaired at seven days postimplantation; all rats had scores of 21 meaning no impairment, or 20 meaning slight trunk instability but otherwise normal function.^[^ ^29^ ^]^ However, to ensure that a longer‐term implantation did not result in decline in motor function beyond this weeklong recovery period, one animal with a bifurcated and one animal with a planar implant were retained for a 5‐week period, and assessed with the BBB‐scale at 14, 21, 28, and 35 days postimplantation. This time‐point was chosen as natural recovery reaches a plateau in rats with thoracic injury at the position of the implant by 5‐weeks.^[^ ^24^ ^]^ Both animals showed no impairment with consistent scores of 21/21 across this extended period (Figure 2d), in contrast to a previous report of moderate hind‐limb deficits at 6 weeks after insertion of a polyimide implant.^[^ ^9^ ^]^ Due to the consistent scoring in these two 5‐week animals we elected to move onto longer‐term implantations, which included an external connector (see Section 2). After perfusion of both the 7 day and 5‐week animals, the implants were observed to still be in position along the spinal cord.

#### 2.2.2 The Bioelectronic Implant Did Not Affect Spinal Cord Volume or Shape

**Figure 3:**
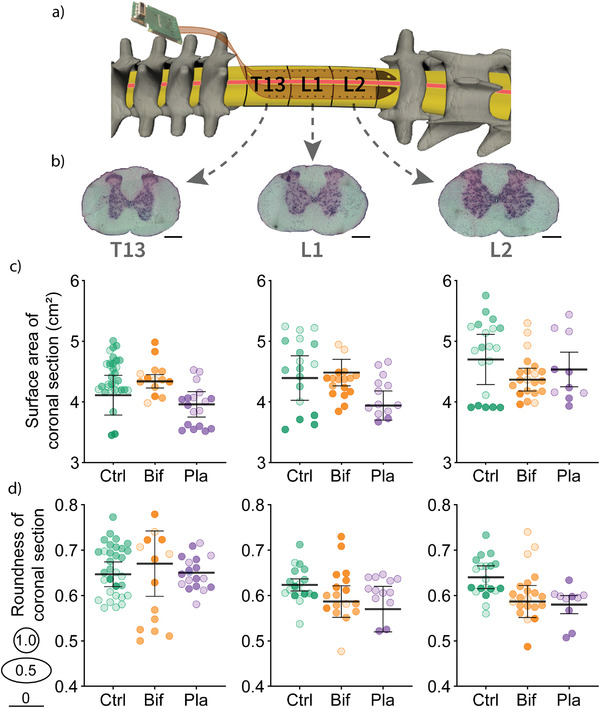
The bioelectronic implant did not significantly impact spinal cord volume or shape. a) Spinal cord segments directly underneath the bioelectronic implant were examined. Note that spinal segment labels do not correspond to the names of the spinal processes above them.^[^ ^30^ ^]^ b) Examples of T13, L1, and L2 coronal sections of spinal cord stained with a hematoxylin and eosin stain to aid with visualization of the tissue are shown (scale bars = 0.5 mm). The perimeter of each section was traced to assess the surface area and shape. c) The surface area of coronal sections is shown, taken from rats without implants (Ctrl), planar implants (Pla) and bifurcated implants (Bif) at the T13 (left), L1 (center), and L2 (right) segments of the spinal cord. d) The shape of sections was also assessed via a roundness score between 1 (circular) and 0 (flat line) at T13, L1 and L2 sections (from left to right) of the spinal cord. Coronal sections are shaded differently for each individual animal on the dot plots, error bars show standard error between animals. Comparisons using one‐way ANOVA revealed no group differences.

Although the bioelectronic implant had no long‐term impact on motor function, we assessed the effect of the bifurcated and planar implants on spinal cord segments directly underneath the implants (T13, L1, and L2) at 7‐days postimplantation (**Figure** **3**a). It is important to note that spinal segment labels do not correspond to the names of the spinal processes positioned directly above them.^[^ ^30^ ^]^ In a previous study, insertion of a 25 µm thick polyimide implant (cf. 8 µm thick implants used in the current study) resulted in a severe deformation in spinal cord volume and shape below the implant.^[^ ^9^ ^]^ We collected coronal spinal sections, stained with hematoxylin and eosin to aid with visualization of the tissue (Figure 3b) from rats implanted with bifurcated (*n* = 3) and planar (*n* = 2) implants for a week and compared them with sections obtained from control rats (*n* = 3), which received the laminectomy only. For each section, the perimeter was traced to obtain the surface area (cm^2^) and shape (roundness).

The average surface area of coronal sections was not significantly different between groups when compared with ANOVA for any of the spinal segments (T13: *F*(2,5) = 0.58, *p* = 0.59, L1: *F*(2,5) = 0.8, *p* = 0.5, L2: *F*(2,5) = 0.3, *p* = 0.75). Coronal segments from the different groups were observed to be similar to each other with no noticeable reduction in size associated with the presence of the implant. However, there was a non‐significant trend for planar implant sections to be smaller at T13 and L1 (Figure 3c).

The shape of spinal cord sections was assessed using a roundness score, which ranged from 1, indicating circular, to 0, indicating a flat line. ANOVAs indicated no significant difference between the groups in the shape of the spinal segments (T13: *F*(2,5) = 0.06, *p* = 0.94, L1: *F*(2,5) = 0.7, *p* = 0.54, L2: *F*(2,5) = 1.25, *p* = 0.26). In general, the shape of spinal coronal sections were similar between groups and there were no malformations as were previously reported.^[^ ^9^ ^]^ However, there was a non‐significant tendency for sections to be slightly less round in the presence of either implant compared with the controls at L1 and L2 (Figure 3d).

We investigated whether deformation of the cord occurred over a more extended period in two of our rats that were implanted for 5 weeks across two spinal cord segments (L1 and L2). After 5 weeks, spinal segments below the implants had an average roundness of 0.68 ± 0.07 at L1 and 0.62 ± 0.03 at L2 compared with the 1‐week control animals, which had roundness scores of 0.62 ± 0.04 at L1 and 0.64 ± 0.04 at L2. The 5‐week implanted rats had rounder L1 spinal segments than controls and shape did not differ at L2. This difference in shape may be related to the increased size and weight of these 5‐week implanted rats compared to the 1‐week controls (≈15% increase in average weight) and we did not compare surface area between these groups for this reason.

#### 2.2.3 Foreign Body Response Related to the Presence of the Implant Was Limited

Implantation of devices on the surface of the brain or spinal cord typically results in a foreign‐body neuroinflammatory response of macrophages (microglia) and astrocytes below the position of the implant.^[^ ^31^, ^32^, ^33^ ^]^ Therefore, coronal sections from two animals per group were immunohistochemically stained with standard markers for astrocytes (GFAP) and microglia (Iba1). In order to focus on changes in these markers, directly associated with the presence of the implants on the dorsal surface of the spinal cord, only the region of spinal tissue directly below the implant was isolated (Figure S1, Supporting Information). Comparison with the same spinal cord segments in control animals, which received the laminectomy surgery but no implant, provided a measure of foreign‐body response associated with the week‐long presence of the bifurcated and planar implants.

**Figure 4:**
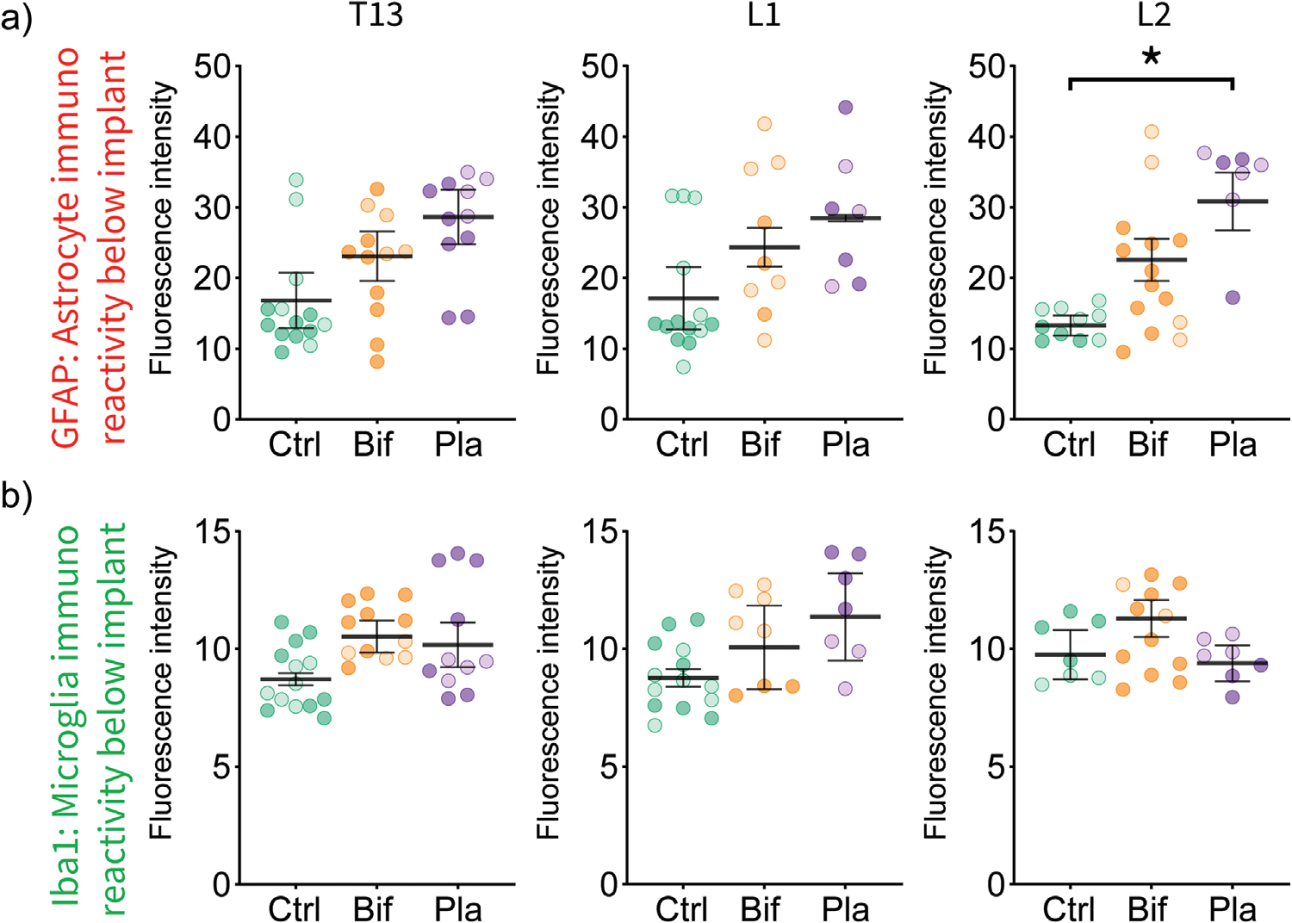
The planar implant had a localized foreign body response of astrocytes compared to controls. After 7 days of implantation, astrocyte and microglia expression was examined in regions of interest (ROI) containing spinal tissue directly below the position of the bioelectronic implant (see Figure S1, Supporting Information). a) The average fluorescence intensity of astrocytes stained with GFAP is shown across the three thoracic segments for the controls (Ctrl), bifurcated (Bif), and planar (Pla) implants. Rats implanted with the planar implant had greater activation of astrocytes compared to the control group in the L2 spinal segment only. b) Rats implanted with either implant did not differ from controls in terms of Iba1 labeled microglia expression. ROIs are shaded differently for each individual animal on the dot plots, error bars show standard error between animals. All comparisons were one‐way ANOVA's, *p < 0.05.

The fluorescence intensity of labeled astrocytes was significantly different between the groups at spinal segment L2 but not at T13 and L1 (ANOVA's: for L2, *F*(2,3) = 16.73, *p* < 0.05; for T13, *F*(2,3) = 2.46, *p* = 0.11; for L1, *F*(2,3) = 3.63, *p* = 0.16; **Figure** **4**a). At L2, the group difference was due to an increased astrocyte response in rats with planar implants compared to control rats (Tukey's post hoc: *p* < 0.05). Florescent intensity of microglia was not significantly different between the groups for any of the spinal segments (ANOVA's: for T13: *F*(2,3) = 1.94, *p* = 0.29), for L1 (*F*(2,3) = 0.75, *p* = 0.54; for L2: *F*(2,3) = 1.33, *p* = 0.39; Figure 4b). The rat kept for 5 weeks with a bifurcated implant had a similar level of astrocyte and microglia florescent intensity as was found at 7 days postimplantation (Figure S3, Supporting Information).

### 2.3 Electrophysiological Spinal Cord Recordings

#### 2.3.1 The Implant and External Plug Housing Remained Viable and in Position for 12 Weeks

**Figure 5:**
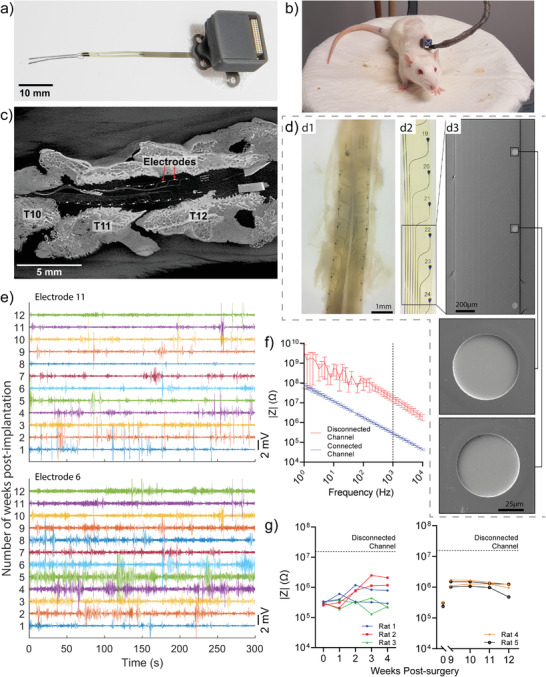
The bioelectronic head and electrodes remained functional and in position along the spinal cord after 12 weeks in rats implanted with a backpack housing for the external plug. a) A resin printed backpack housed the printed circuit board and plug. A circular piece of surgical mesh was attached to the ventral surface of the backpack, which was positioned subcutaneously forming a strong attachment to the tissue. b) The backpacks allowed rats to be plugged in via a recording cable while sitting and ambulating upon a raised platform. c) After 12 weeks, a cylinder of tissue was excised, containing the intact spinal cord around the bioelectronic head of the implant, and micro‐CT scanned to ascertain the position of the implant. An overhead view of the bioelectronic head shows that the electrodes still oriented correctly along the surface of the spinal cord (red arrows point to two electrodes). The scour marks in the spinal bone where the T10, T11, and T12 spinal processes were removed are visible, showing the implant is still in the expected rostral‐cranial position. d) After 12 weeks the bioelectronic implant was removed and electrode surfaces imaged with SEM. d1) Light microscope image of the implant after removal from the animal with small amounts of tissue remaining. d2) Light microscope image of the implant after cleaning. d3) SEM image of the implant and two electrodes highlighting the undamaged electrode surfaces after 12 weeks of implantation. e) Spinal cord electrical activity was recorded while the rat was on the raised platform, weekly recordings from two different electrodes are shown from the same rat. f) Characteristic impedance of a connected electrode channel and a disconnected electrode channel, measured in vitro before implantation. Dotted line indicates 1 kHz, where the in vivo impedance is quantified. g) In vivo impedance measurements at 1 kHz for two cohorts of rats over 0–4 and 9–12 weeks. The dotted line indicates the impedance that would be expected from a disconnected channel at 1 kHz.

Ten rats had bifurcated bioelectronic implants inserted along the thoracic spinal cord, and backpack assemblies (**Figure** **5**a) were attached to the back muscles via sutures and surgical mesh. Animals were connected two or three times per week for 3–5‐min recording sessions, during which they ambulated and sat upon on a raised platform (Figure 5b). Backpacks remained viable and firmly attached to the back muscle and skin of the animals over a 12‐week period. A 12‐week implantation time was chosen with future experiments in mind, to have a sufficient window to both apply and then evaluate the benefits of regenerative treatments for spinal cord injury delivered via the implant. Two of the animals were tested in the open field using the BBB‐scale, and both scored 21/21 at 1, 3, and 7 days after surgery, and every week after that, up to 12 weeks postimplantation. To examine implant positioning, one of the animals was perfused at 89 days postimplantation. The backpack and outer muscle was carefully removed, and the bioelectronic body of the implant was severed, leaving the implant head in position along the spinal cord. A cylinder containing the intact spinal cord and including ≈1 cm of tissue at every side was dissected and processed for micro‐CT scanning. When the scanned images were reconstructed, a dorsal “bird's eye” view showed both arms of the implant still positioned appropriately along the spinal cord inside the laminectomy (Figure 5c), which confirmed that the implant had not been displaced over the 12‐week period. The metallic tracts and electrodes were also intact after the long implantation period.

To further validate that the integrity of the electrodes and tracts would remain 12 weeks postimplantation, we examined explants from two rats using scanning electron microscopy (SEM). For this analysis, adherent tissue first had to be removed from the probes (Figure 5d1) after which the cleaned sample could be analyzed, first under light microscopy (Figure 5d2) and then at higher magnification using high resolution SEM (Figure 5d3). We could confirm that electrode tracts and electrodes were intact and found no signs of delamination (Figure 5d3). This underlines the high biostability of our polyimide‐based probes, especially considering that the extensive cleaning necessary to remove adherent tissue and prepare the probes for imaging puts additional stress on the material. Furthermore, to examine functionality of the electrodes in vivo, live impedance of several electrodes per animal was recorded from weeks 1–4 and 9–12 postimplantation in five rats. Prior to implantation, each electrode's impedance spectrum was similar across the entire range of 10 kHz to 1 Hz when tested in salt solution (0.01 m PBS) (Figure 5f, “connected channels”). In contrast, and as expected, much higher resistance was seen across the entire frequency range in channels not connected to an electrode (Figure 5f, “disconnected channels”). Weekly impedance measures of electrodes in the freely behaving rats were collected using the same protocol used during the saline test. At 1 kHz, all electrodes displayed impedance well under what would be expected for a disconnected channel (Figure 5g). Week‐to‐week impedance variation was evident but with a tendency toward slow increase, which is expected for this kind of live impedance setup.^[^ ^34^ ^]^ Over the time course of our experiments, none of the electrodes displayed the impedance of a disconnected channel which, together with the SEM explant analysis, further supports that our implants remain intact and functional.

#### 2.3.2 Spinal Cord Electrical Activity Was Recorded in Freely Moving Rats

Recordings of spinal cord electrical activity were taken 2–3 times a week throughout the full study period of 12 weeks in three rats. The recordings were made as the rats moved around a raised platform and consisted of a range of activities including walking, sitting, rearing, and grooming. The parallel rows of electrodes on the bifurcated arms are positioned along the surface of the spinal cord at the border of the inside arch of the dorsal horn and the dorsal column, a region containing both neurons and bundles of axons involved in relaying somatosensory signals from the hind‐feet and hind‐region of the body to the brain.^[^ ^35^ ^]^ Figure S1A (Supporting Information) illustrates the approximate position of the electrodes shown as small black rectangles embedded in the orange line which represents the implant.

The electrical recordings remained viable over the 12‐week time‐course. Figure 5e shows an example of weekly recordings from two single electrodes on the left bifurcated arm of the implant; which was positioned on the right hemisphere of the spinal cord above spinal segment T13 (electrode 11) and L1 (electrode 6). Additional weekly recordings from different electrodes in two rats are shown in Figure S4 (Supporting Information).

**Figure 6:**
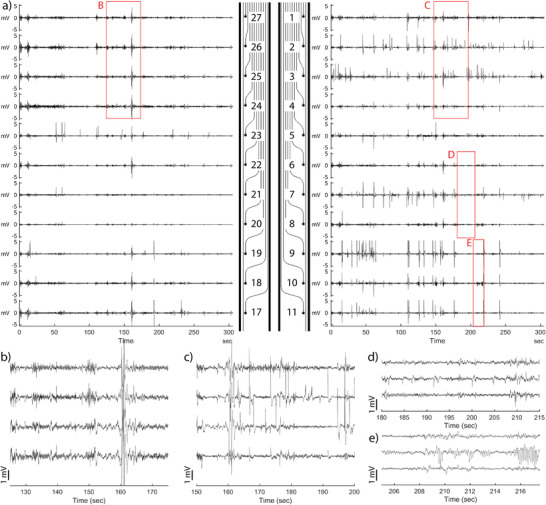
Electrical activity patterns varied across electrodes during recording sessions. a) Concurrent electrode activity is shown for the left and right arms of the bioelectronic implant while the rat ambulated on a raised platform for five minutes. The relative activity varies across electrodes, and voltage spikes vary greatly in magnitude. Numbers on the implant diagram correspond to the electrode ID. Magnified voltage traces from adjacent electrodes highlighting characteristic voltage waveforms. b) Highly correlated voltage changes on adjacent electrodes that are potentially indicative of motion artifacts. c–e) Voltage spikes that are isolated to specific channels, indicative of physiological electrical changes. Scale bar = 1 mV.

**Figure** **6** shows electrical activity from every electrode during a single recording, which was taken 7 days after implantation, the same time point used for the histology in the earlier experiments. The electrical activity is shown for the right and left arms of the bioelectronic implant. Within this recording session, the amount of activity varied across all of the electrodes, and a higher general level of activity is noticeable on one of the implant arms (right side of Figure 6).

Figure 6b–e shows magnified voltage traces of electrical activity from adjacent electrodes. While it is clear that some changes in voltage are indicative of recording artifacts, the highly localized nature of voltage spikes suggests physiological electrical activity. Figure 6b shows voltage changes that are strongly correlated with neighboring channels as is often characteristic of motion artifacts in in vivo electrode array recordings. In contrast, Figure 6c shows short isolated voltage spikes that are not correlated between neighboring channels, suggesting high spatial localization. These voltage spikes vary in magnitude with a few higher amplitude spikes evident in the range of 1–3 mV, whereas the majority of signals have an amplitude below 2 mV. These differences in spike magnitude may be related to varying distance between source and electrode.^[^ ^36^ ^]^ Similarly, Figure 6d shows lower amplitude voltage spikes that are localized to individual channels. Figure 6e shows repetitive voltage spikes that reach between ≈1 and 2 mV below the baseline and oscillate in rapid succession. Additional examples showing all electrode activity during a single session from different animals and time‐points are shown in Figures S5–S9 (Supporting Information).

## 3 Discussion

This work details the fabrication and characterization of an innovative subdural bioelectronic implant designed to be positioned above a spinal cord injury to monitor changes in electrical activity and capable of acting as a delivery platform for electroceutical and chemical treatments. Insertion of the internal parts of the implant in rats had no effect on physiological function or spinal cord volume and shape, at least over the 5 weeks studied here. Foreign body response to the presence of the implant was only detectable when the planar design of the implant was used, and only localized to one segment of the spinal cord. Next, we designed an external housing for the implant capable of being surgically attached to the upper back muscle for a period of up to 12 weeks, after which we were able to show that the bifurcated implant remained in position along the spinal cord. SEM analysis of explants after and live impedance readings suggest that the electrodes and tracts remain viable over 12 weeks of implantation. Lastly, we demonstrate electrical recordings captured from the device in awake, freely moving rats.

The bioelectronic implant is constructed of a thin flexible polyimide, which is a highly durable material that shows excellent compatibility with nervous tissue, which previously has been demonstrated for intracortical and epicortical microdevices.^[^ ^6^, ^12^, ^16^, ^17^, ^37^, ^38^, ^39^ ^]^ Beyond fundamental biomaterials properties, biocompatibility of a bioelectronic implant is a matter of mechanical properties in the context of the intended in vivo location of the implant, which in turn is a combination of material mechanics and the geometry of the device itself.^[^ ^40^ ^]^ A direct comparison to biocompatibility of devices within different tissues is therefore not possible, even if made from the same thin‐film polyimide, stressing the importance of performing this evaluation specifically for the spinal cord. This point is further emphasized by a previous study, which showed that a polyimide implant inserted along the lumbosacral spinal cord in rats resulted in significant hind‐limb motor deficits 6 weeks after surgery.^[^ ^9^ ^]^ Minev et al. found that polyimide implanted rats made significantly more errors on a horizontal ladder task than sham rats, and rats implanted with a PDMS implant. Furthermore, a comprehensive analysis of hind‐limb gait revealed that polyimide implanted rats had significantly impaired foot control in terms of step height, orientation, and hip joint amplitude, as well as reduced leg movement, and lateral foot placement compared to sham and PDMS implanted rats. This suggests that these rats would have scored around 12–15 on the BBB‐scale. These are strong arguments in favor of a transition toward even softer and elastomeric materials than polyimide. Nevertheless, we argue that an alternative modification should be considered, based on our previous work with epicortical grids. In the study by Vomero et al.^[^ ^16^ ^]^ it was clearly shown how a polyimide‐based implant can go from non‐conformable to conformable (with respect to the brain surface) simply by reducing the thickness of the polyimide itself, and how this in turn was decisive for their biocompatibility. While non‐conformable implants resulted in depression of the cortex, the versions that were conformable induced minor tissue response and little impact on the shape of the cortical surface. We suggest these results are analogous to the 8 µm implants shown here, as the spinal cord preserved its rounded shape. Furthermore animals in the current study, in contrast to the study by Minev et al., showed only some mild transient hind‐limb impairments 1–3 days after surgery, with no impairment after 1 week and the following weeks up to 5 weeks postimplantation. In the current work we found no differences in spinal cord volume or shape in rats implanted for one or 5 weeks compared to the severe deformation of the spinal cord in rats implanted with the thicker and wider planar shaped polyimide implant after 6 weeks.^[^ ^41^ ^]^ We argue that the different outcomes in these studies are most likely explained by differences in the design of the polyimide implants used where the implant described by Minev et al. was just over three times thicker (25 vs 8 µm) and almost twice as wide (3.2 vs 1.85 mm) as the bioelectronic implants described here. It should be noted that in previous experiments we implanted wider and thicker versions of our bioelectronic implant in rats (2.15 mm width, 12 µm thick), which resulted in hind‐limb deficits in some animals (data not included). It is also important to point out that in Minev et al., implants were positioned further back (spinal segments L2 to S1) and covered twice the length of spinal cord than in the current study (3 vs 1.5 cm). We are convinced that the width, thickness and design of the implant play a key role in its tendency to cause damage to the spinal cord environment and result in physiological impairment whilst implanted. Thus, the difference in mechanical properties between the elastomer PDMS and polyimide are here compensated by the latter allowing for an ultra‐thin design, which ensures structural biocompatibility of the resulting implant. Considering the challenges associated with manufacturing of stretchable implants,^[^ ^11^ ^]^ it is highly encouraging that structural biocompatibility appears to be possible also with non‐stretchable but flexible polymers and conventional thin‐film metallizations.^[^ ^11^ ^]^ An additional shortcoming of thin‐film devices is their higher sensitivity to degradation, both because their overall small volume allows little tolerance for degradation and because of inherent stress in thin‐film layers may result in delamination unless layers are strongly bound.^[^ ^42^ ^]^ We here report on the stability of our thin‐film devices, as demonstrated by in vivo impedance spectroscopy and high resolution imaging of explants, which shows that also this challenge can be tackeled by thin‐film technology.

We did not detect an increase in the presence of microglia in the region of spinal cord directly below the bioelectronic implant, although there was an increase in astrocytes in the L2 region for the planar design of the implant only. This difference may be a result of the planar style implant straddling the posterior spinal cord vein which is positioned above the surface of the spinal cord in the subdural space, whereas the bifurcated arms are positioned either side. Therefore, the planar device may exert more pressure upon this central vein^[^ ^43^ ^]^ and would not conform as well to the spinal cord surface on either side. It is likely that the bifurcated implant design avoids these issues in the same way that highly fenestrated polyimide‐based epicortical devices have been shown to conform better to surface of the brain.^[^ ^16^ ^]^ For this reason we used the bifurcated design exclusively for the longer‐term electrophysiology experiments. Foreign body response due to the presence of the bioelectronic implant may be more pronounced over a longer time‐span of implantation,^[^ ^32^ ^]^ which will be investigated in future work.

The backpack housing, positioned just below the shoulder blades on the upper back, is a modified versions of the lower back housings developed by Wurth et al. for sciatic nerve implants.^[^ ^13^ ^]^ The backpack has several advantages over skull mounting, which is used in brain neural recording devices,^[^ ^44^, ^45^ ^]^ and has also been used for spinal cord implants.^[^ ^9^, ^19^ ^]^ The use of the backpack allows for a less invasive surgery, as the device is positioned just in front and above the laminectomy in the same incision site used for insertion of the bioelectronic implant. This can be useful when combining the implant with a mild or moderate spinal contusion injury model, in which many changes in physiological function due to natural recovery occur in the first week after injury, which coincides with surgical recovery. Skull‐mounted devices use micro screws embedded in acrylic cement to make a strong attachment, and the dorsal skull plates are often removed with the device. Our bioelectronic implant is designed to deliver electrical treatments to an injury site to regenerate damaged neural tissues, after which the implant could be removed. Unlike a skull‐mounted device, the backpack can be surgically removed once recovery is achieved. However, the viability of the backpack anchoring method will need to be further tested in spinal cord injured rats and over the planned experiment time (12 weeks) with the inclusion of frequent recording and stimulation sessions, which will put additional strain on the device. We envisage that 12 weeks is sufficiently long for treatments to influence regeneration and any resulting functional improvements to be observed in a rat model of SCI.^[^ ^24^ ^]^

In vivo electrophysiological recordings can be contaminated by noise and artifactual signals that affect subsequent analysis and interpretation of the signal. Artifacts may be of extrinsic origin, such as a noisy electromagnetic environment or the measurement instrument itself, or they may be of intrinsic or physiological origin, such as non‐neural bioelectrical signals. Artifact removal in this work was not performed beyond the removal of 50 Hz line noise and the subtraction of a common average reference channel (Figure S2, Supporting Information). In contrast, physiological artifacts, such as those from non‐neural bioelectrical signals, are more complicated to remove. In the more established field of EEG common physiological artifacts arise from electromyograph (EMG) signals from muscle activity, in particular the heartbeat, eye movements and blinks.^[^ ^46^ ^]^ A comprehensive understanding of physiological artifacts present will require correlation or electrophysiological recordings with movement behavior and sensory stimulation as well as peripheral nerve stimulation under anesthesia to establish ground truth signals.

The bioelectronic implant has tremendous potential for future use in testing treatments for SCI, both electroceuticals (electric field therapies) and as a drug delivery platform capable of applying regenerative treatments directly to the injured spinal cord for sustained periods. SCI is a devastating condition that can result in permanent neurological impairment, and there are currently no regenerative therapies available.^[^ ^47^ ^]^ However, promising approaches to achieve axonal regeneration have been identified in vitro, such as the application of electrical fields.^[^ ^21^ ^]^ Weak electric stimulation (up to 80 mV mm^–1^) has been shown to enhance axonal regeneration as well as guide orientation in vitro.^[^ ^23^ ^]^ However, a major challenge is to apply treatments directly to a SCI and over a continuous treatment period. Although not explicitly addressed in this work, the subdural polyimide bioelectronic implant we describe here is suitable for providing spatiotemporally precise electric field generation delivered via larger dedicated stimulation electrodes. As the fabrication follows standard protocols for thin‐film bioelectronics, functional electrode materials such as nanostructured platinum, iridium oxide or PEDOT/PSS could be added in a few additional steps according to previously described protocols.^[^ ^26^ ^]^

Here we have shown spinal cord electrical recordings in freely moving rats for the first time, over a period of 12 weeks. In future work we plan to characterize this activity over a 12 week period and compare uninjured control rats with SCI rats, and SCI rats treated with daily electrical stimulation across the injury site. Subtle changes in electrical activity after SCI may provide early indications that treatments are working. Furthermore, using electrical recordings to map the boundaries of an injury site could also have additional value in a clinical setting to detect the extent of an injury and inform personalized electroceutical treatments.

## 4 Conclusion

We have demonstrated a bioelectronic implant, which can be safely implanted along the spinal cord for a clinically relevant period. In contrast with previous reports of attempts at subdural polyimide implants, the implant reported here has no deleterious effect on motor function and only minimal effect on spinal cord shape and foreign body response. The very thin polyimide based implant was able to conform to the cord and the embedded microelectrodes recorded, for the first time, highly localized voltage spikes and electrical activity from the spinal cord in freely moving rats over 12 weeks. This device has great potential to monitor electrical signaling in the spinal cord after an injury and will facilitate the identification of biomarkers of injury and recovery. In the future this platform could also be used to deliver localized electroceutical treatments for spinal cord injury.

## 5 Experimental Section

### Bioelectronic Implant Fabrication

Two different polyimide device designs were fabricated comprising a (i) planar 1.85 mm width and (ii) bifurcated 1.85 mm width polyimide device (Figure 1). These widths were chosen as the diameter of the spinal cord was ≈3 mm. The bioelectronic devices were fabricated using standard microlithography techniques in the cleanroom facility at University of Freiburg (RSC, ISO 4, according to ISO 14644‐1). First, U‐Varnish‐S polyimide (PI, UBE Industries, Ltd., Japan) was spun on silicon wafers (at 3000, 4500, and 9000 rpm for achieving an after‐annealing thickness of 6, 4, and 2 µm, respectively). Then, tracks/interconnection lines, connection pads and active sites were patterned using the high resolution image reversal photoresist AZ 5214 E (MicroChemicals GmbH, Germany). O_2_ plasma (80 W, Plasma System 300‐E, PVA TePla, Germany) was used to activate the surface and as an adhesion promoter for the metal, which was subsequently evaporated onto each wafer (100 nm of Pt, Univex 500 Electron‐Beam Evaporator, Leybold GmbH, Germany). Lift‐off to remove excess metal was done in acetone. O_2_ plasma was again used to activate the surface of the wafers before a second PI layer was spun onto all of them. The positive photoresist AZ 9260 (MicroChemicals GmbH, Germany) was used as a masking layer for the opening of the contact pads and electrode sites and for setting the outlines of the devices. PI was finally etched using O_2_ plasma in a reactive ion etching (RIE) machine (STS Multiplex ICP, SPTS Technologies, United Kingdom) with a multi‐step protocol (200 W first and 100 W after, time varying with PI thickness). The photoresist was stripped off in an acetone bath and the devices were mechanically peeled off from the wafers using flat tweezers.

### In Vitro Electrochemical Characterization

Initial characterization of the implants included characterization of all electrodes by electrochemical impedance spectroscopy (EIS) and cyclic voltammetry (CV) measurements in a phosphate buffered saline (PBS, 0.01 m) electrolyte. An electrochemical workstation (Biologic) with a three‐electrode set‐up was utilized and comprised a silver/silver chloride (Ag/AgCl) reference electrode, platinum counter electrode and the respective platinum electrode to be tested on the bioelectronic device as the working electrode. For CV measurements, potential limits were set at 0.9 and −0.6 V versus Ag/AgCl and each electrode was scanned three times at a scan rate of 100 mV s^–1^. EIS measurements were carried out at the electrodes open circuit potential and utilized a 50 mV sinusoidal potential ranging from 10 000 to 1 Hz. The slope of abs(*Z*) was derived by dividing the difference in *y*‐coordinates of 2 points on the line by the difference of the *x*‐coordinates of those same 2 points.

### In Vivo Biocompatibility Experiments

Ten female Sprague‐Dawley rats, between 210 and 258 g at time of surgery, were obtained from the Vernon Jansen Unit, University of Auckland in compliance with the University of Auckland Animal Ethics Committee Guidelines (AEC002056) and the New Zealand Animal Welfare Act 1999. Animals were housed together in a dedicated colony and behavioral room on a 12:12 light/dark cycle and always had ad libitum access to rat chow and water. They were group housed prior to surgery, after which they were individually housed.

### Surgical Procedure

Animals were anesthetized with isoflurane, temperature monitored and placed on a heating pad and given subcutaneous antibiotics (Baytril 100 mg mL^–1^, 0.1 mL per 100 g) and analgesics (Carprofen 50 mg mL^–1^, 0.01 mL per 100 g; Bupivicaine 2.5 mg mL^–1^ 0.16 mL per 100 g; Buprenorphine 0.3 mg mL^–1^, 0.17 mL per 100 g). A laminectomy was performed to expose the spinal cord at the spinal processes T10, T11, and T12. Small incisions in the dura were made near either end of the exposed spinal cord. A guidance catheter, containing a suture anchored to the tail of the implant, was inserted below dura at the T10 end and carefully propelled along the surface of the spinal cord, exiting at the T12 end. The suture was then used to guide the implant along the subdural space and used to position the implant so that the bioelectronic head was in contact with the spinal cord. The bifurcated design of the bioelectronic used two guidance catheters (one attached to either arm) which were used to guide and position the arms either side of the posterior spinal cord vein. Once the implant was positioned, the guidance suture was removed. A thin piece of absorbable gelatin sponge (Pfizer Gelfoam, Amtech Medical Limited, Wanganui) was used to form a hemostatic seal in the space above the exposed spinal cord. At the tail end, the implant was connected to a small PCB which routed the electrode channels to a 36‐channel Omnetics female nano‐strip plug. In some animals, the implant was briefly connected via a recording cable to MEA2100 system to make test recordings. The protruding section of implant near the printed circuit board was then severed. The deeper muscle tissue was sutured above the Gelfoam using an absorbable suture (ChomicGut 4.0) and the skin was sutured closed using nylon suture (Ethicon 4.0, Johnson and Johnson) so that no part of the implant was subcutaneous or external to the animal. This directly tested any effects of the presence of the implant along the spinal cord without extraneous factors, an approach adopted by others during development of a sciatic nerve implant.^[^ ^48^ ^]^

### Postoperative Care

Postoperatively, animals were given subcutaneous antibiotics (Baytril 100 mg saline^–1^, 0.1 mL per 100 g), analgesics (Carprofen 50 mg mL^–1^, 0.01 mL per 100 g; Buprenorphine 0.3 mg mL^–1^, 0.17 mL per 100 g), and supplementary fluid (saline, 3 mL) twice daily for 3 days. Bladder function was assessed, however, it was not necessary to manually void the bladder on any of the rats.

### Assessment of Locomotor Function

The BBB locomotor rating scale was used to measure right and left hind‐limb motor function. Animals were placed in a circular open field (100 cm diameter white wooden floor with 20 cm Perspex walls) in the recovery room. Following the surgery, rats were tested on postoperative day's 1, 3, and 7 for 5–8 min each time depending on the amount of movement exhibited. This length of time has been determined as providing sufficient time to observe and record behavioral recovery of individual rats with minimal risk of missing key findings. Two of the animals were retained for 5 weeks to examine how hind‐limb function was effected by longer‐term implantation. They were assessed with the BBB‐scale on days 1, 3, 7, 14, 21, 28, and 35 postsurgery. BBB‐scores were determined by a single experimenter (BH) blinded to the condition of the rats during scoring. The functionality of each hind‐limb was scored from 0 (total paralysis) to 21 (normal movement) as described previously,^[^ ^29^ ^]^ and the scores for the right and left hind‐limbs averaged.

### Histological Sectioning

At the end of the 1 or 5‐week behavioral assessment period, animals were euthanized with sodium pentobarbital (100 mg kg^–1^, i.p.) and perfused intracardially with 0.9% saline (400 mL) followed by 4% paraformaldehyde (400 mL, in 0.1 m phosphate buffer, pH 7.4). ≈15 mm of spinal cord with the 10 mm extent of spinal cord that contacted the bioelectronic head of the implant in the center was dissected, removed and post fixed in 4% paraformaldehyde for 4 h then transferred to 20% sucrose and stored at 4 ˚C. After 3–4 days the cord was transferred to 30% sucrose containing 0.1% sodium azide. Spinal cords embedded in OCT compound were sectioned coronally (from caudal to rostral) at 10 µm using a Cryostat and mounted onto positively charged glass slides.

### Hematoxylin and Eosin Staining

The majority of slides were stained with a hematoxylin and eosin (H&E) stain to aid with visualization of the tissue for the assessment of shape and surface area of the spinal cord. Briefly, sections were hydrated and then immersed in hematoxylin solution, differentiated in acid alcohol, color‐shifted toward blue using Lithium Carbonate, and then cross‐stained with Eosin Y solution. Between these steps, slides were placed under running tap water for several minutes. Slides were then rehydrated using graded ethanol, deparaffinized in xylene and mounted and coverslipped using DPX.

### Immunohistochemical Staining

The remaining slides were stained with two standard cellular markers for foreign‐body response of astrocytes (GFAP, red) and microglia (Iba1, green). Immunohistochemistry was carried out using the following antibodies; mouse anti‐glial fibrillary acidic protein (GFAP)–Cy3 (Sigma, 1:2000) to show astrocyte structure, and goat anti‐Iba1 (Abcam, ab5076, 1:250) to label microglia. Antibodies were diluted with 4% normal donkey serum (NDS) in PBS–T and incubated overnight at 4 °C. Control sections on each slide were GFAP only, Iba1 only, and NDS only (no primary antibody). The next day, slides were washed in PBS and incubated with a 1:250 dilution of donkey anti‐goat Alexa‐488 (Iba1) in 4% NDS in PBS–T for 4 h at room temperature. The nuclei of cells were counterstained using Hoechst 33342 (Thermo‐Fisher Scientific #62249). Slides were washed with PBS and coverslip mounted using ProLong Gold Antifade Reagent (Invitrogen). Staining was done in batches, which each included a mix of slides from different experimental animals and groups.

### Imaging and Analysis

Tiled imaging of whole H&E‐stained coronal sections was performed using a 10× objective on an EVOS M7000 microscope. Immunohistochemically stained whole sections were imaged using a 4× objective on an EVOS M5000 microscope to ensure no distortion of fluorescent intensity due to tiling. Fluorescent images were taken using the appropriate filter sets (RFP, GFP, DAPI) and all microscope settings were kept consistent. Imaged sections were aligned using the midline as an anatomical landmark and the filenames blinded by a colleague who was not part of the study. A maximum of three sections per spinal cord block were imaged per slide. Each coronal section was cross‐referenced with a Rat Spinal Cord Atlas^[^ ^30^ ^]^ to determine vertebral location along the spinal cord, and Harrison et al. (2013) was used to determine the corresponding spinal cord segment. The perimeter of each H&E‐stained section was traced in ImageJ and the surface area (cm^2^) and shape (“roundness”) measured. Roundness used the formula: 4 * area/(*π* * major_axis^2^). A value of 1.0 indicated a perfect circle, whereas decreasing values indicated an increasingly elongated shape with a value of 0 indicating a flat horizontal line. For immunohistochemically stained sections, analysis was restricted to a region of interest of tissue directly below the implant position (2.4 mm centered across the dorsal surface and 200 µm deep. The mean pixel intensity of fluorescence in this region was determined in ImageJ for the GFAP and Iba1 images from each section.

### Electrophysiological Recording Experiments

Ten female Sprague‐Dawley rats, between 210 and 290 g at time of surgery, were obtained and housed in the same room and conditions as described previously.

### Bioelectronic Backpack Assemblies

The polyimide implants were carefully soldered to pads on a small custom printed circuit board (10 × 12 mm) which routed the electrodes, two grounds, and reference to a 32‐channel Omnetics female nanostrip connector soldered into drill hole vias on the other side and end of the board. Fine silk suture (Ethicon, 7.0) was inserted into a 1.5 cm piece of 28 G polyurethane rat intrathecal catheter (Alzet), looped through an ear‐hole at the tip of the implant arm, inserted back through the catheter, and then snipped and superglued at the tip of the catheter. The backpack was 3D printed in grey resin using a Creality LD‐002R Resin 3D printer at 50 µm resolution. An ≈2 cm diameter section of surgical mesh was attached to the ventral surface of the backpack with non‐absorbable nylon suture (Ethicon 4.0) through 4 small slots in the midline body of device and through the hole at the apex of each of the five legs. The polyimide implant was carefully dropped through a small internal slot in the backpack and the PCB board and Omnetics connector positioned within the square upper section of the backpack. The components were then secured with a lid inserted into grooves on the dorsal surface and filled with epoxy resin glue. Figure 5a shows a completed bioelectronics backpack assembly. Electrochemical impedance spectroscopy (as described below) was used to test whether each electrode channel was valid (see Live Impedance Readings).

### Surgical Procedure

In these animals, bifurcated bioelectronic implants were inserted as described above. Once the Gelfoam had been inserted and the deeper muscle sutured closed, the backpack and surgical mesh were positioned in a pocket beneath the skin just above the cranial end of the incision and in front of the laminectomy site. The body of the bioelectronic implant protruded from the deeper muscle below the surgical mesh at the rostral end of the assembly and was carefully folded back on itself below the assembly to provide slack. The five legs protruding from the ventral surface of the backpack were then sutured to the deeper muscle through the surgical mesh using non‐absorbable nylon suture (Ethicon 4.0). Over several weeks, the mesh provided a scaffold for tissue regrowth resulting in a strong anchor ensuring minimal risk of the implant or backpack becoming detached. The skin was sutured closed tightly around the base of the backpack using silk suture (Ethicon 4.0, Johnson and Johnson), and the hind‐limb claws were carefully trimmed under the microscope to reduce damage caused by scratching.

### Electrical Recording Sessions

Rats were acquired 3 weeks before the surgery date, over which they were habituated to handling by the experimenters for at least 5 days per week. After several days of handling, the rats were habituated to sitting and ambulating upon a raised platform (130 cm height, ≈32 cm diameter) over several weeks. During postsurgery recording sessions, rats were placed on the platform with the recording cable hanging overhead. The backpack was then gently grasped by an experimenter to aid in connection (and removal) of the recording cable plug. Once connected, the cable hung loosely and did not restrict the movement of the animal. Recordings were then made for a duration of 3–5 min during which animals were awake and allowed to freely behave on the platform.

### Live Impedance Readings

An electrochemical workstation (Biologic) with a three‐electrode set‐up was connected to the implant via the recording cable. Impedance spectra was measured at the electrodes open circuit potential with a 100 mV sinusoidal potential from 10 000 to 1 Hz, with 10 points per decade logarithmic spacing. The reference was connected to a larger electrode (0.157 mm^2^) positioned in the center of the implant at the point where the arms bifurcated. The counter was connected to shorted electrodes in the tip of each bifurcated arm (0.630 mm^2^). The working channel was connected to the electrode being tested. Prior to implantation, the bioelectronic head of the implant was immersed in a phosphate buffered saline (PBS, 0.01 m) electrolyte. Impedance spectra of all electrodes was measured and compared with “disconnected” channels, which ended at the implant PCB and were not connected to an electrode. Connected electrode channels had an impedance of ≈5.4–5.6 MΩ at 1000 Hz, whereas disconnected channels were ≈7.2–7.4 MΩ at 1000 Hz (Figure 5f). It should here be noted that in vivo impedances were collected in a three‐electrode configuration, with the large electrode positioned at the center of the implant as reference, and the two large electrodes at the tip of each bi‐furcated arm as counter electrodes. Thus, the measurement conditions are different from the ideal in vitro measurements, and do not serve to accurately evaluate the impedance of each electrode in vivo. Nevertheless, it is possible to clearly differentiate between disconnected and connected channels, as shown in Figure 5f, meaning the recorded impedances would reveal any broken electrodes. Live impedance was measured using the same setup in three rats from weeks 1 to 4 and two other rats from weeks 9–12 post‐implantation (Figure 5g). These different time‐points are provided in the different sets of animals due to COVID‐19 lockdown and social distancing policies in New Zealand. These recordings were made using the same setup as for electrical recordings except with the electrochemical workstation connected to the recording cable.

### Explant Analysis and Imaging

A bifurcated probe was explanted together with a short section of the spinal cord for subsequent analysis of the probe itself. Light microscopy was used to document the position as well as status of the probe while still being attached to the explanted spinal cord segment. All adherent tissue was subsequently removed using tweezers and a scalpel blade to expose the actual polyimide probe for detailed light microscopy analysis. The cleaned explant was finally coated with a thin layer of gold (8 nm, CCU‐010, Safematic) for high resolution SEM imaging of individual electrode sites (Helios 5CX, ThermoFischer).

### Recording Equipment and Signal Processing

Electrical recordings were acquired with a Multi‐Channel Systems ME2100 (Harvard Bioscience) electrophysiology rig. The raw analog signals were amplified ten‐times and acquired with a sampling frequency of 20 kHz per channel, which was converted to a digital signal at a resolution of 24 bits. Raw data were converted to HDF5 format and then further processed in MATLAB (2018b, Mathworks) using in‐house scripts. Line noise from the power supply was removed using a 50 Hz notch filter. While it is common to apply bandpass filters to electrophysiological recordings to isolate specific data at specific frequencies, the relevant frequency ranges for spinal electrical activity are not well established, and consequently the data are presented without bandpass filtering. Rather, a median channel was then calculated and subtracted from each individual channel. The effect of the median channel subtraction can be seen in Figure S2 (Supporting Information). Specifically, the median channel contains changes in voltage that are common to each electrode, and therefore likely artifactual. By subtracting the median, the processed data reflect changes in voltage that are unique to an individual, or a small number, of electrodes. The median subtraction was done separately for the electrodes of each arm of the implant.

### Statistical Analysis

Statistical analysis was performed in GraphPad Prism. Where relevant, sample sizes were reported in the text. Data are presented as mean ± standard error, except where noted in the text. Error bars were represented the standard error. *P*‐values were calculated using one‐way ANOVA with a Tukey post hoc test carried out across groups and without any preprocessing by transformation, removal of outliers, or normalization. In all cases, significance was defined as *p* ≤ 0.05.

## Conflict of Interest

The authors declare no conflict of interest.

## Supporting information

Supporting Information

## Data Availability

The data that support the findings of this study are available from the corresponding author upon reasonable request.
