# Supplementary material for: A Subdural Bioelectronic Implant to Record Electrical Activity from the Spinal Cord in Freely Moving Rats
Source: Adv Sci (Weinh). 2022 May 2;9(20):2105913. doi: 10.1002/advs.202105913 (PMC9284137; doi:10.1002/advs.202105913)
Supplement: Supplementary file 1 — Supporting Information [file ADVS-9-2105913-s001.pdf]

Supporting Information

A Subdural Bioelectronic Implant to Record Electrical Activity from the Spinal Cord in Freely Moving Rats

*Bruce Harland, Zaid Aqrawe, Maria Vomero, Christian Boehler, Ernest Cheah, Brad Raos, Maria Asplund, Simon J O'Carroll and Darren Svirskis**

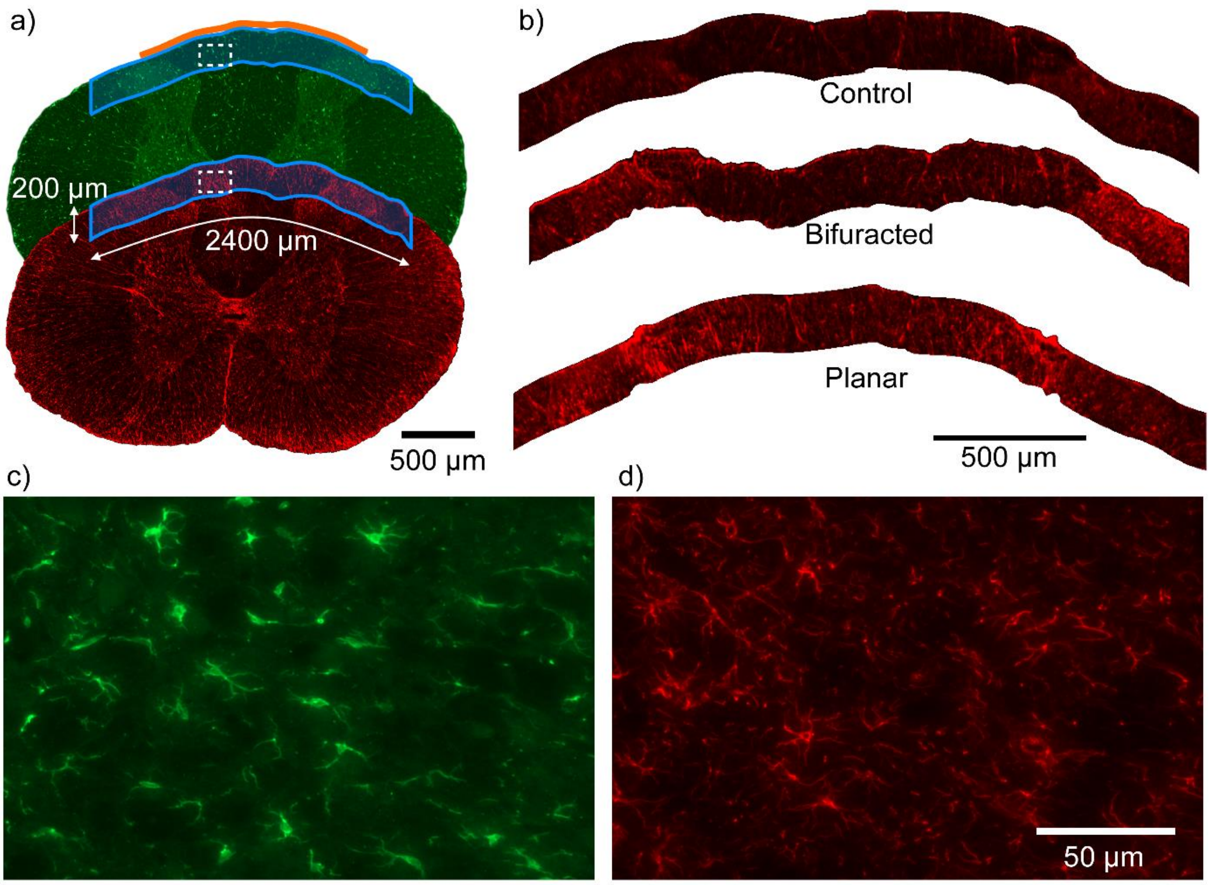

Figure S1. The neuroinflammatory response of astrocytes and microglia was examined in spinal tissue directly below the position of the bioelectronic implant. a) Coronal spinal cord sections were stained with standard cellular markers for astrocytes (GFAP; red) and microglia (Iba1; green). The approximate position of the bioelectronic implant is shown by the orange line at the top of the image (not to scale). For each section, only the region of interest (ROI) of spinal cord tissue directly below the implant was isolated and analysed (blue boxes). Sections shown are from a rat implanted with planar implant. b) Representative examples of ROI's

from each group stained with GFAP to label astrocyte-response (example ROI's are from spinal segment T13). Examples of c) microglia stained with Iba1, and d) astrocytes stained with GFAP are shown at 40x magnification, these images were taken from the white dotted box indicated in panel A.

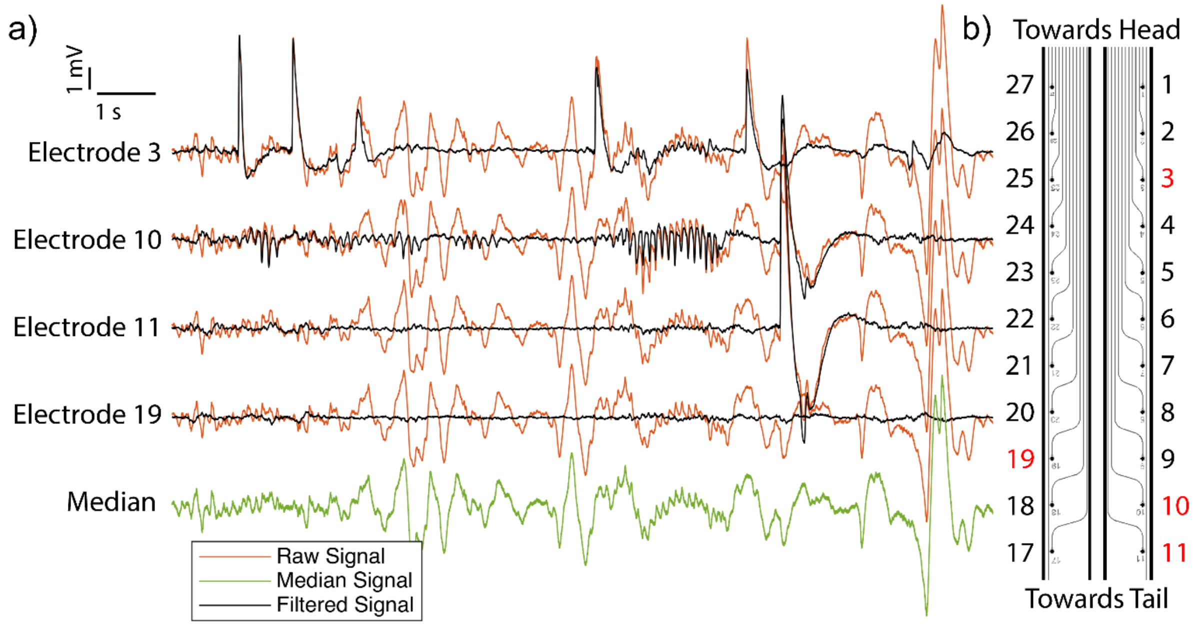

Figure S2. The effect of median channel subtraction on the electrical recordings. Data corresponds to electrodes 3, 10, 11 and 19, shown in Figure 6, between 214 – 220 seconds. a) Raw, median and filtered signals for four individual electrodes, highlighted in b) in red. The median channel contains changes in voltage that are common to all channels. Subtracting the median channel from each individual channel reveals electrical activity that is unique to a single or a small number of individual electrodes.

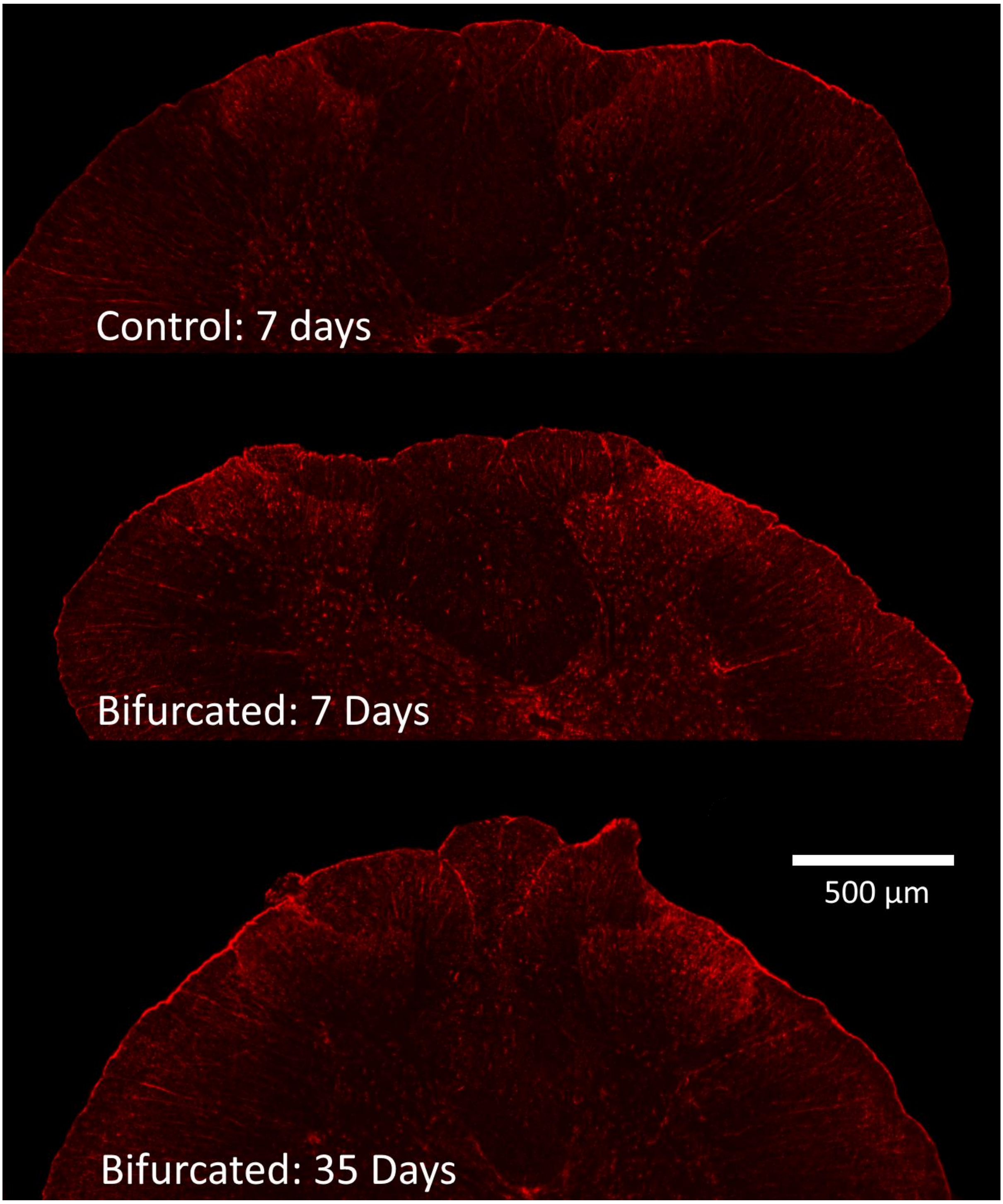

Figure S3. Astrocyte response after 5-weeks was similar to 1-week in rats with bifurcated implants**.** Examples of T13 spinal segment coronal sections stained with GFAP are shown from a control rat (top), and rats with bifurcated implants after 1 week (middle) and 5-weeks (bottom). Although not quantitative, this is highly suggestive that foreign body response stays at a tolerable level up to 5-weeks, which is consistent with the finding that the 5-week animal had no hind limb functional impairment.

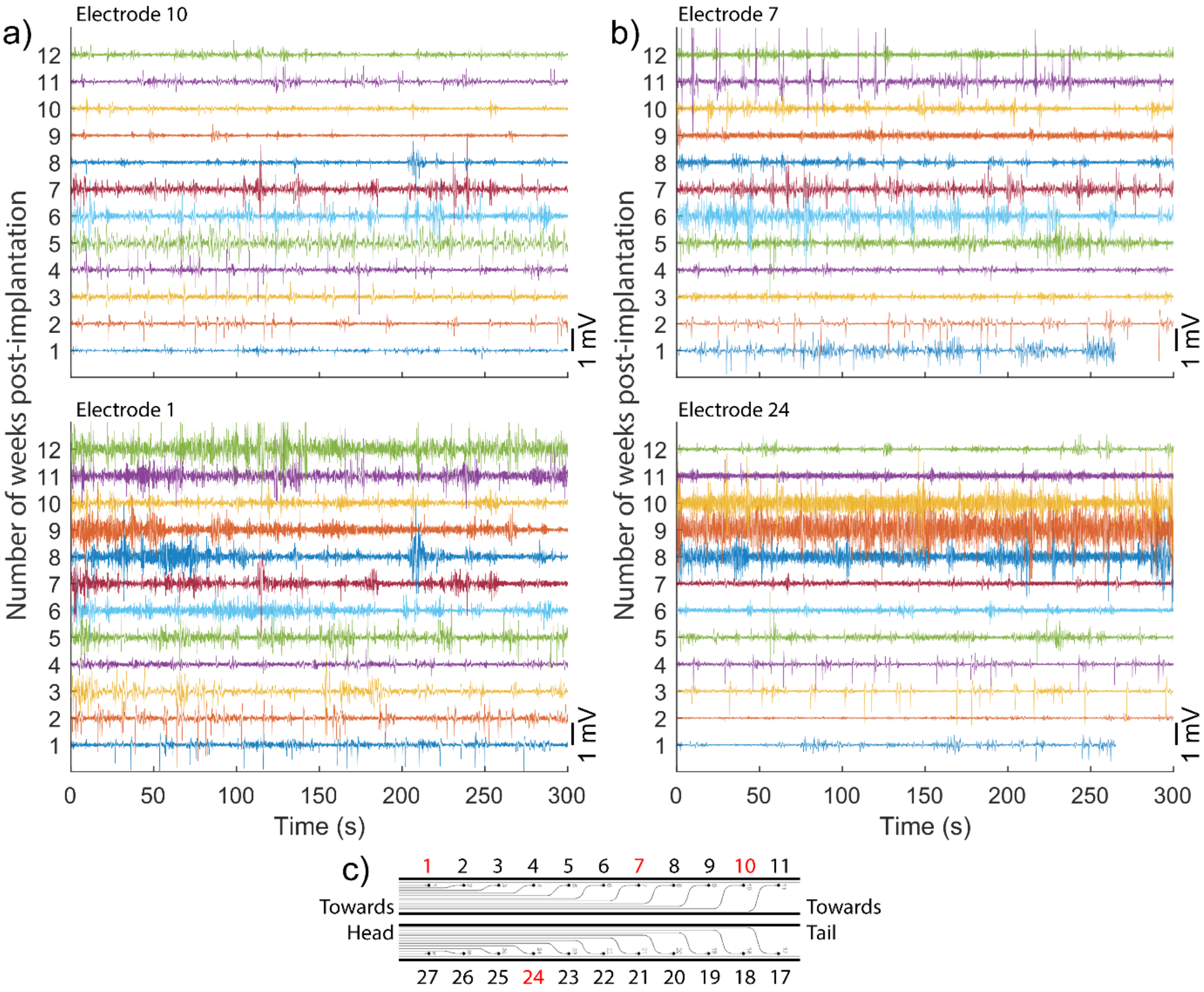

Figure S4. Additional examples of longitudinal weekly spinal cord recordings from two animals while the rat was on the raised platform. Within a) and b) two different electrodes are shown from each animal. c) Schematic of the implant indicates the electrodes shown above, highlighted in red text.

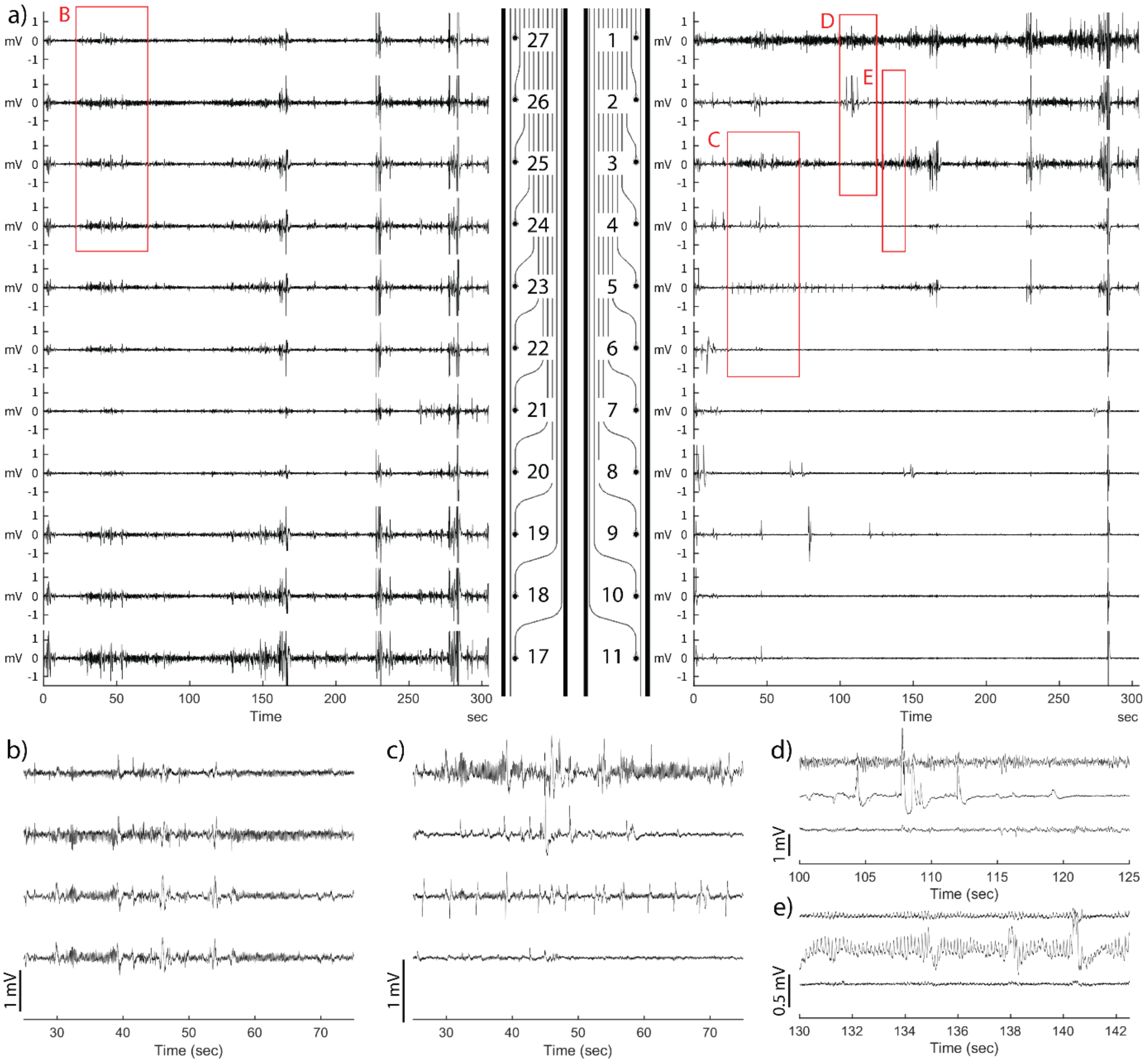

Figure S5. Example of electrical activity across all electrodes within a single recording session. Data corresponds to the same animal as shown in Figure 6 at 68 days post-implantation. a) Overview of all data recorded within the session. b, c, d, e) Magnified voltage traces from adjacent electrodes highlighting characteristic voltage waveforms.

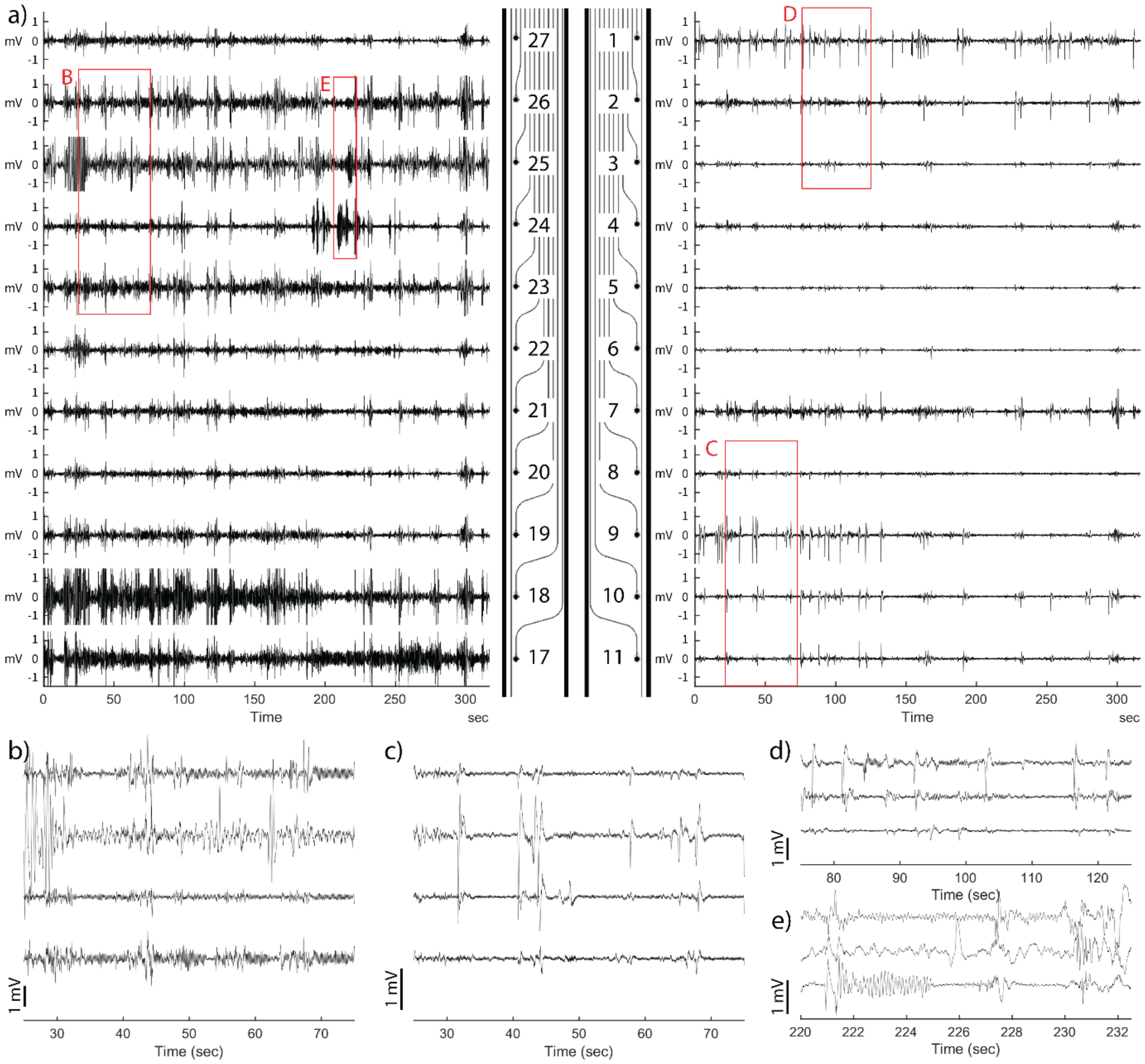

Figure S6. Example of electrical activity across all electrodes within a single recording session. Data from a second animal at 10 days post-implantation. a) Overview of all data recorded within the session. b, c, d, e) Magnified voltage traces from adjacent electrodes

highlighting characteristic voltage waveforms.

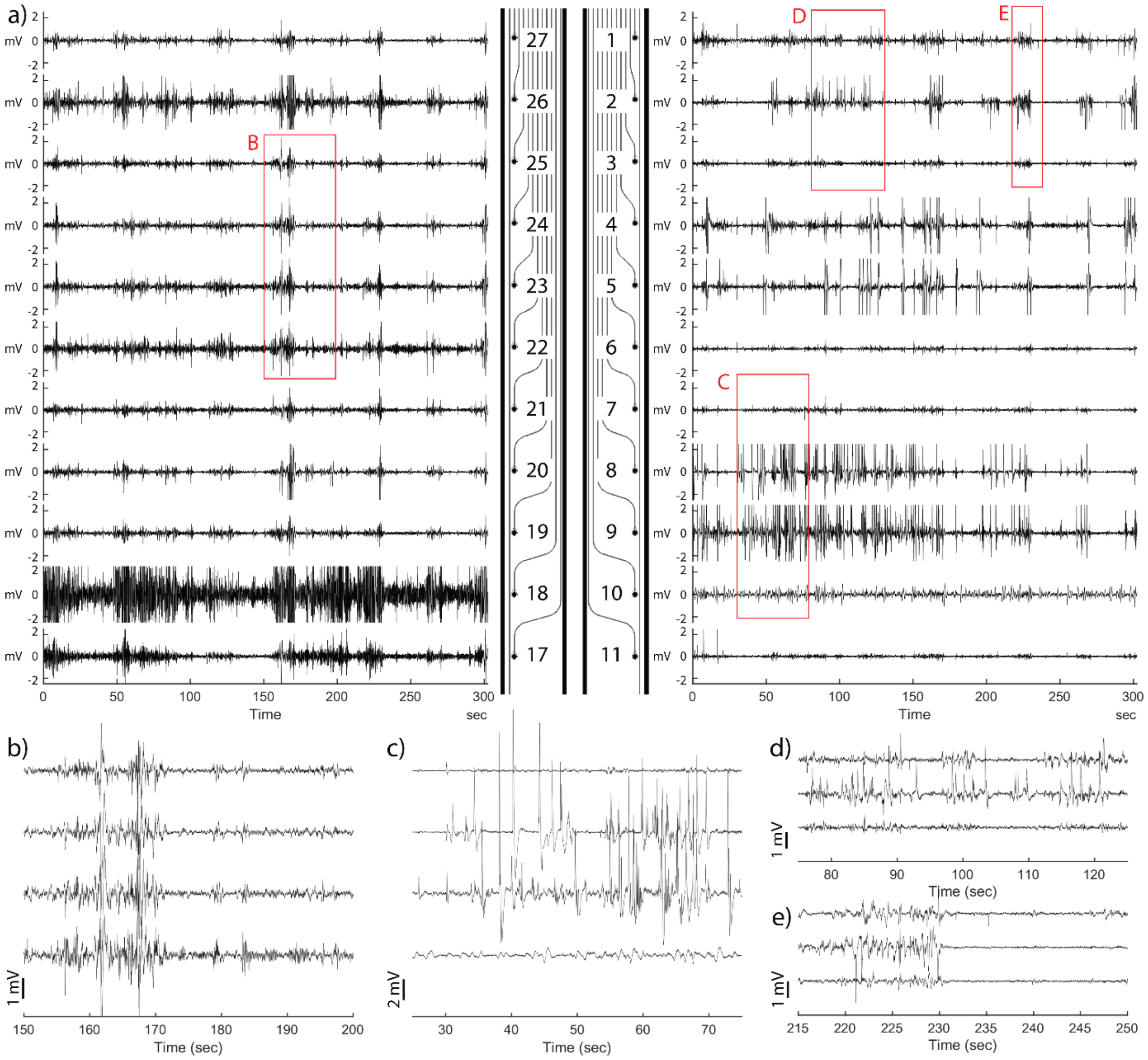

Figure S7. Example of electrical activity across all electrodes within a single recording session. Data from a second animal, corresponding to Figure S6, at 34 days post-implantation. a) Overview of all data recorded within the session. b, c, d, e) Magnified voltage traces from adjacent electrodes highlighting characteristic voltage waveforms.

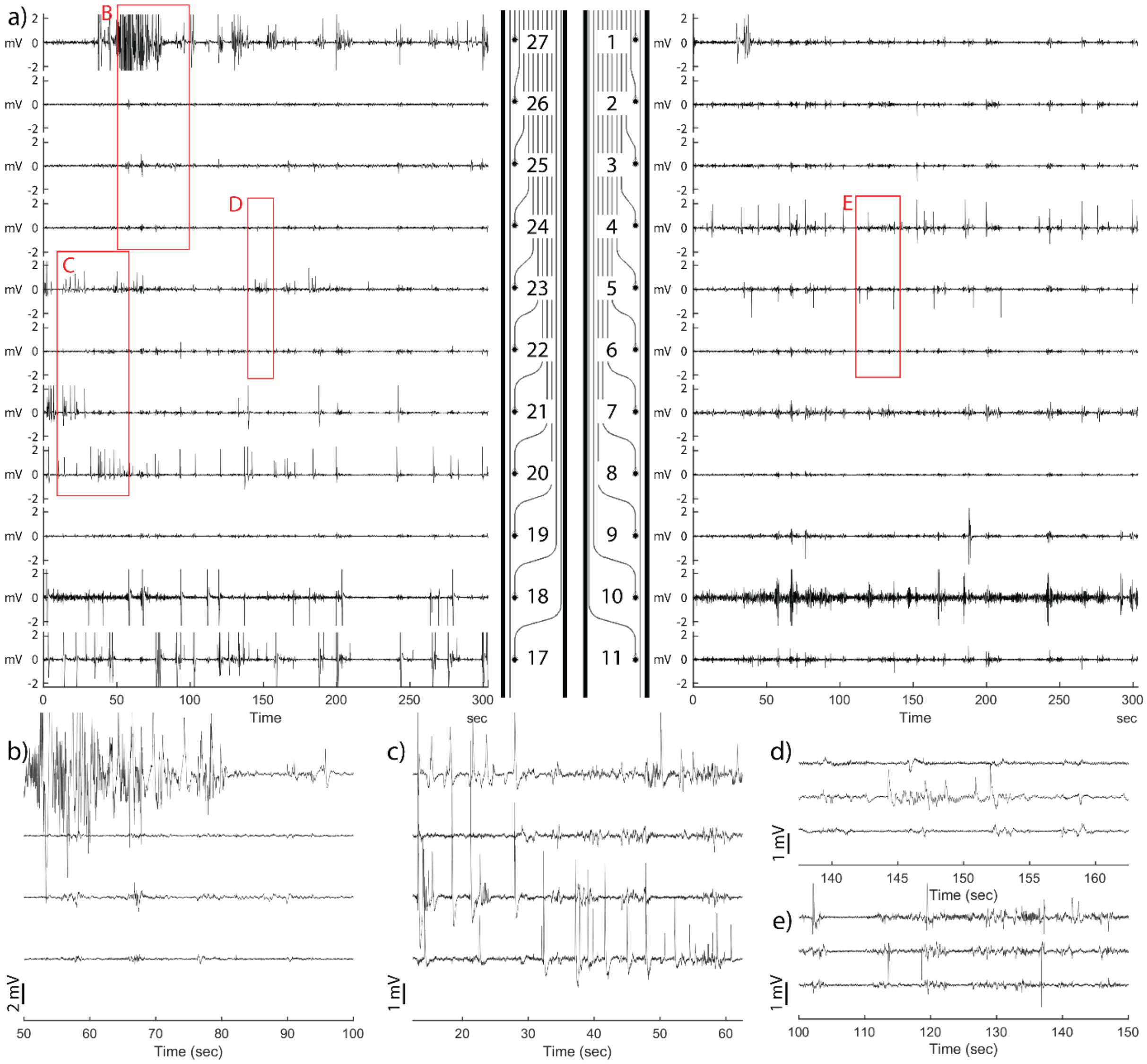

Figure S8. Example of electrical activity across all electrodes within a single recording session. Data from a third animal at 28 days post-implantation. a) Overview of all data recorded within the session. b, c, d, e) Magnified voltage traces from adjacent electrodes highlighting characteristic voltage waveforms.

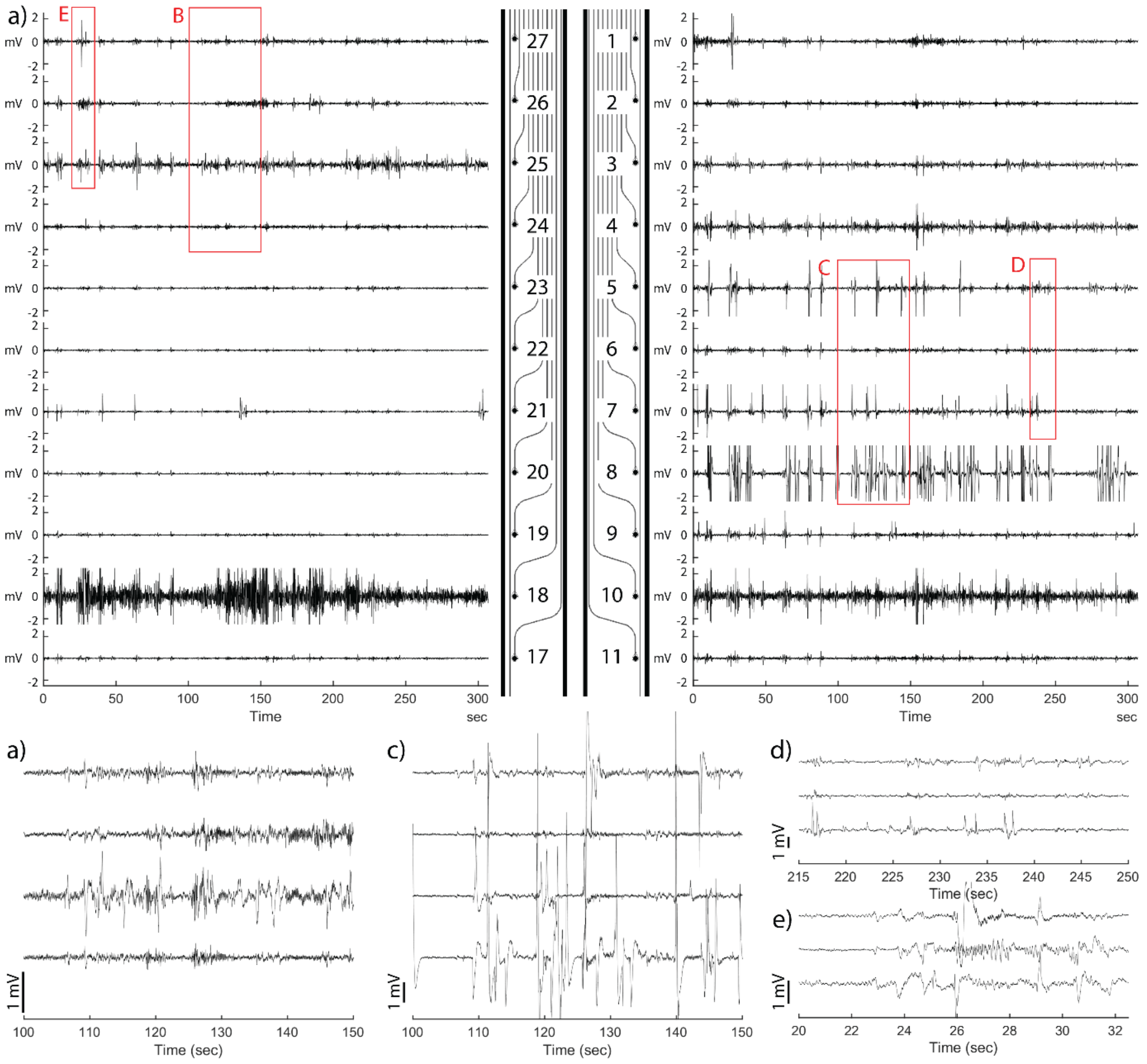

Figure S9. Example of electrical activity across all electrodes within a single recording session. Data from a third animal, corresponding to Figure S8, at 56 days post-implantation. a) Overview of all data recorded within the session. b, c, d, e) Magnified voltage traces from adjacent electrodes highlighting characteristic voltage waveforms.
